# Effects of Maximal Concentric Contractions on Myotendinous Mechanical Properties in Prepubertal Boys

**DOI:** 10.1002/ejsc.70212

**Published:** 2026-09-17

**Authors:** Baptiste Chanel, Nicolas Babault, Carole Cometti

**Affiliations:** ^1^ Centre d’Expertise de la Performance, Faculté des Sciences du Sport Université Bourgogne Europe Dijon France; ^2^ Université Bourgogne Europe INSERM, CAPS UMR 1093 Dijon France

**Keywords:** children, fatigue, Growth, shear wave elastography

## Abstract

This study aimed to compare the effects of a fatiguing concentric exercise on the myotendinous mechanical properties of the knee extensors between men and prepubertal boys. Ten prepubertal boys and 10 men performed a fatiguing exercise consisting of five sets of 20 maximal isokinetic concentric knee extensor contractions. Before the exercise, the mechanical properties of the vastus lateralis (VL), the rectus femoris (RF), and the patellar tendon (PT) were assessed using shear wave elastography to measure the shear wave velocity (SWV) at 70° of knee joint flexion for PT and 70° and 110° for VL and RF. The tests also included maximal voluntary isometric (MVIC) and concentric (MVCC) contractions. These measurements were repeated after the exercise (Post), 30 min after (Post30), and 48 h after (Post48 h). Significant main time effects were observed for SWV VL 70° (*p* = 0.007) and RF 70° (*p* < 0.001), with lower values at Post compared to Pre. No age effect or interaction were observed for VL and RF SWV 70°. For SWV PT, a main age effect was observed, highlighting lower values for boys (*p* < 0.001), without a time effect or interaction. A significant decreased MVIC was observed for men at post, returning to baseline at Post30, whereas no changes in MVIC were observed in boys. These findings suggest that, under acute conditions, maximal concentric contractions induce comparable changes in myotendinous mechanical properties between prepubertal boys and men, while men exhibit a greater force production decline.

## Highlights

- A concentric exercise protocol (5 x 20 maximal contractions) did not affect tendon mechanical properties in prepubertal boys or men.
- Exercise‐induced changes in muscle mechanical properties were comparable between boys and men.
- Maximal voluntary isometric torque declined in men but was preserved in boys following exercise.

## 1 Introduction

Repeated contractions during physical training can affect the mechanical properties of the myotendinous complex, reducing both force production and transmission (Zhou 1996). In adults, tendon properties may be impaired, as evidenced by a decrease in tendon stiffness after repeated isometric (Kubo et al. 2001) and eccentric contractions (Obst et al. 2016; Yin et al. 2014). After eccentric exercises, muscle mechanical properties have also been observed, as highlighted by an increase in rectus femoris (RF) shear wave velocity (SWV) (Heales et al. 2018; Lacourpaille et al. 2017). This increase in muscle SWV is commonly attributed to the presence of muscle damage (Ličen and Kozinc 2022), caused by the important fascicle strain (Doguet et al. 2019). In contrast, after isometric contractions, a decrease of VL SWV has been observed (Siracusa et al. 2019; Chalchat et al. 2020). It has been attributed to an elevation of the intramuscular temperature and a reduction and the force capacity of the cross‐bridge caused by fatigue (Chalchat et al. 2020). Altogether, these results highlighted deleterious acute effects for both eccentric and isometric contractions on the myotendinous mechanical properties.

While eccentric and isometric contractions have been explored, less is known about the effects of concentric contractions on myotendinous mechanical properties. Since prepubertal children have been shown to exhibit a high capacity to maintain force production during concentric exercises (Chanel et al. 2025), this supports the relevance of investigating this exercise modality in the pediatric population. However, despite this apparent resistance to fatigue, less is known about the alterations in myotendinous mechanical properties. It may be hypothesized that concentric exercises induce limited perturbations of the myotendinous structure because it does not involve active lengthening of the myotendinous complex and is generally associated with lower torque production. Nevertheless, this assumption remains largely untested, and several factors may to contribute to exercise‐induced changes in myotendinous elastic properties (Siracusa et al. 2019; Chalchat et al. 2020). This concern is particularly relevant given the poor efficiency of children in the stretch‐shortening cycle and force transmission capacity (Radnor et al. 2018). It is reasonable to suppose that an acute modification of their myotendinous properties may exacerbate their reduced mechanical efficiency, thereby affecting some fundamental capacities (i.e., sprinting and jumping). Moreover, the period of growth is associated with the emergence of pathologies involving the myotendinous structures (Hansen et al. 2023). Therefore, it is crucial to identify and manage the effects of exercise on the myotendinous mechanical to prevent excessive mechanical loading on developing structures. Previous studies have shown that children are less susceptible than adults to muscle damage following eccentric or plyometric exercises (Chen et al. 2014; Marginson et al. 2005). However, it has also been reported that myotendinous complex stiffness, assessed during isometric ramp contraction, was decreased after isometric contractions, regardless of the age group (Piponnier et al. 2020). Thus, the difference between boys and men in the acute modifications of myotendinous mechanical properties appears to be exercise‐dependent.

To optimize physical training prescription, it is crucial to identify clearly the myotendinous mechanical alterations in various exercise modalities. Given that concentric contraction mode has not been well explored, this study will focus on this exercise modality. Considering the literature, this may not impair tendon properties and may be associated with a fast recovery or the torque production capacity (Dundon et al. 2008). The results of this research would be helpful for practitioners to improve physical training periodization by minimizing the mechanical loading on developing structures in prepubertal boys.

Therefore, the main aim of this study was to compare acute changes in the knee extensors' myotendinous mechanical properties induced by concentric exercise in men and prepubertal boys. The hypothesis was that concentric exercise would induce a decrease in the muscle shear wave velocities but would not have an effect on tendon properties, regardless of the age group.

## 2 Materials and Methods

### 2.1 Participants

A total of 10 boys and 10 men (Table 1) were recruited for this study. None reported lower limb injuries or musculoskeletal diseases. They were involved in at least 3 hours per week of sports activities in a competitive context (handball, rugby, basketball). All participants were informed about the purpose of the study and the experimental procedure. The boy's somatic maturity was assessed using the Mirwald formula to calculate the age from the peak height velocity (APHV) (Mirwald et al. 2002). To be included, the APHV of the participant had to be greater than the real age. The self‐indication of pubic hair development was also used to estimate the pubertal stage using the Tanner stages (Tanner and Whitehouse 1976).

**TABLE 1:** Participants characteristics.

Variable	Boys (*n* = 10)	Men (*n* = 10)
Age (years)	10.8 ± 1.1	23.7 ± 2.2
Height (cm)	146.8 ± 8.0	176.7 ± 8.3
Mass (kg)	32.9 ± 3.8	71.7 ± 14.2
APHV (years)	13.6 ± 0.6	—
Difference with APHV (years)	−2.8 ± 0.7	—
Tanner stage (pubic hair)	1–2	—
Physical activity weekly (hours)	4.5 (± 1.1)	6.6 (± 3.4)

*Note:* Values are means ± standard deviation. APHV: Estimated age to the peak height velocity. No significant difference (*p* = 0.089) was observed in weekly training volume between prepubertal boys and men (boys: handball *n* = 4; track and field *n* = 2; rugby *n* = 1; basketball *n* = 1; soccer *n* = 2/men: soccer *n* = 3; track and field *n* = 2; tennis *n* = 1; rugby *n* = 1; volleyball *n* = 1; basketball *n* = 2). None of the participants were specifically resistance‐trained, and none of the men reported participating in more than two resistance‐training session per week.

The sample size was determined a priori using the software G*Power (Version 3.1.9.6), considering the SWV as the main outcome criterion, with the following criteria: effect size of 0.42, power of 0.8, and probability of error of 0.05 (Chanel et al. 2026). A sample size of 10 participants per group was indicated. This study was conducted according to the Declaration of Helsinki and was approved by the local ethics committee. The participants were informed about the nature of the study, and they signed (legal representative for boys) an informed consent.

### 2.2 Experimental Design

To complete the protocol, the participants came to the laboratory twice, at the same hour of the day, separated by 48 h. The first session consisted of the concentric exercise and the tests. The second session included only the post tests. The tests were composed of SWV measurements, maximal voluntary isometric (MVIC), and concentric (MVCC) contractions. They were realized in the first session, before (Pre), immediately after the exercise (Post), after 30 min of recovery (Post30 min), and during the second session (Post48 h). The tests and the exercise were conducted on the right side, ignoring the side dominance, due to logistical constraints related to the experimental setup. Participants were all familiar with the isokinetic contractions with an ergometer thanks to previous participations in physical tests during training or other experimental studies.

### 2.3 Data Collection

During the sessions, the participants were seated on an ergometer (Biodex 4 Quickset, Biodex Corporation, Shirley, NY, USA) with an 80° hip angle (0°: full hip extension) with their lateral femoral condyle aligned with the rotation axis of the ergometer. They were strapped on the pelvis and the trunk to avoid disturbing movements. The standardized warm‐up started with five isometric contractions with an increasing intensity to reach the maximum at the last one. Then, they realized two sets of eight maximal concentric contractions, separated by 1 minute.

Then, Pre‐tests (SWV, MVIC, and MVCC) were realized. The SWV measurements were performed with an Aixplorer Ultrasound scanner (Supersonic Imagine, Aix‐en‐Provence, France) with a linear probe (Linear 10–2). The probe was positioned approximately as follows: 25%–50% for RF, 50%–66% for VL (0%: greater trochanter; 100%: lateral femoral condyle) (Heales et al. 2018), and in contact with the distal part of the Patella for PT. The probe was positioned longitudinally to the muscles and tendon, in the central part of their belly for VL and RF. The optimal probe positions were determined with the B‐mode and were marked on the skin to ensure measurement consistency. For each site, two 8‐s videos were recorded in SWE mode, with the opacity map fixed at 0% to ensure blind data collection. For VL and RF, the elasticity scale was fixed at 8 m.s^−1^ and 14.1 m.s^−1^ for PT (Heales et al. 2018).

The measurements for VL, RF, and PT were realized with a 70° knee joint flexion (Heales et al. 2018). The muscles' properties were also assessed with a 110° knee joint angle. This configuration has been shown to be more sensitive to reveal changes in the muscle properties following fatiguing exercise (Lacourpaille et al. 2017). These joint angles were selected to facilitate comparisons with previous studies and to minimize the risk of measurement saturation that may occur during the assessment of tendon properties at long myotendinous lengths (Chanel et al. 2025). To ensure the absence of muscular contraction during the SWV measurements, electromyographic activities (EMG) of VL and RF were recorded. Two pairs of silver chloride surface electrodes were placed over the muscle belly after skin shaving and cleaning with alcohol. A root mean square value higher than the mean + 3 × standard deviation observed a posteriori of the session was considered as an exclusion criterion. No participant was excluded.

The force tests were conducted next (approximatively 3 minutes after the fatiguing exercise) to avoid any bias effect of the contractions on the SWV. First, two 5‐s MVIC of the knee extensors were executed with the knee flexed at 70°, allowing a 15‐s rest between them. Secondly, after one minute of recovery, three MVCC were performed (from 100° to 20° of knee flexion at a speed of 60°.s^−1^).

The fatiguing protocol consisted of five sets of 20 maximal voluntary concentric contractions of the knee extensors, with 90 s of recovery separating each set. They were realized with the same range of motion and speed as concentric contractions during the MVCC tests. The participants were blinded from torque output.

### 2.4 Data Analysis

**FIGURE 1:**
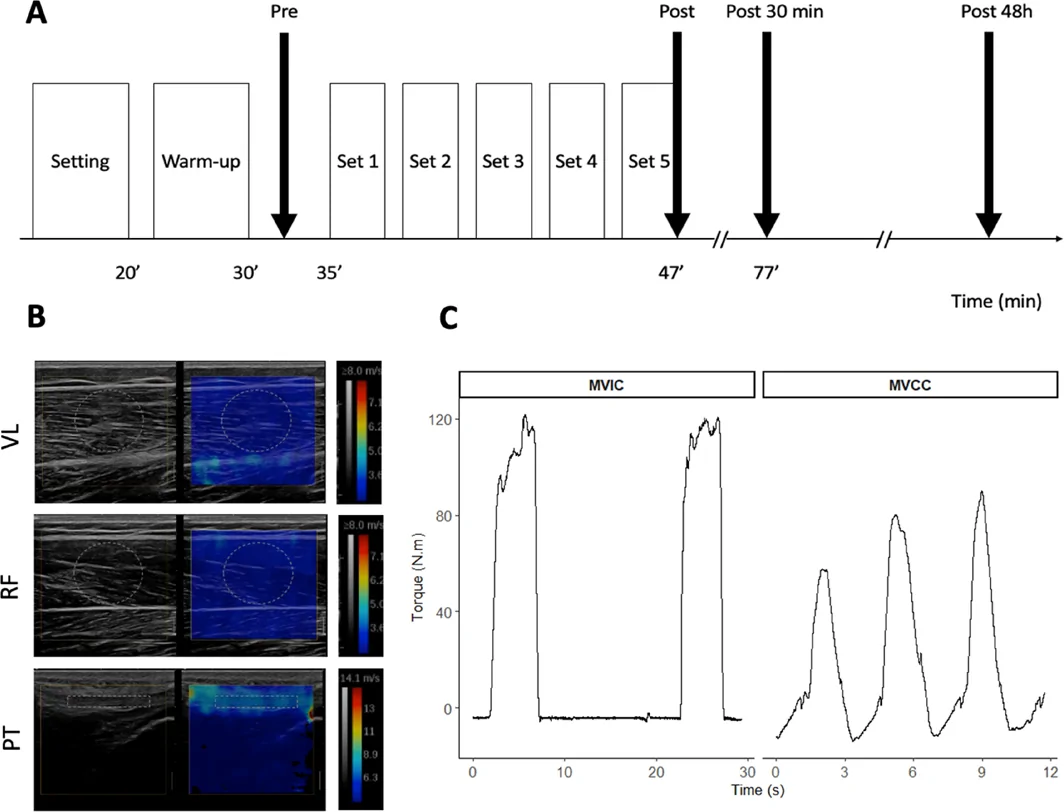
A: Experimental design. Setting corresponded to the placement of the volunteer on the ergometer and the localization of the echography sites. The warm‐up consisted of isometric and dynamic contractions. Each set of the fatiguing protocol was composed of 20 maximal voluntary eccentric contractions of the knee extensors. The tests were composed of shear wave velocity (SWV) measurements for the vastus lateralis (VL), the rectus femoris (RF), and the patellar tendon (PT), two maximal voluntary isometric contractions (MVIC), and three maximal voluntary concentric contractions (MVCC). B: Region of interest used for muscles and tendon investigation. The first line represents an acquisition for VL, the second one is RF, and the third is the PT. The B‐mode and the shear wave elastography are presented in the left and right columns, respectively. The areas considered for the SWV are represented by the white circle for the two muscles and by the white rectangle for the patellar tendon. C: Typical boy's torque data for knee extensors MVIC and MVCC.

Analysis of the SWV data was conducted using the echograph software. The mean of the SWV values of the two videos was used for analysis (6 images extracted per video). For VL and RF, the region of interest (ROI) was as large as possible, excluding muscle aponeurosis (Wang et al. 2022) (Figure 1). For PT, the ROI was the central part of the patellar tendon and covered approximately 75% of the distance between the distal part of the patella and the tibial plateau (excluding parts close to the bone's insertions).

Torque during isometric and dynamic contractions, and the EMG during SWV measurements were collected using a Biopac MP150 system and associated software (AcqKnowledge 4.2 for MP systems, Biopac System, Santa Barbara, CA, USA) at a 1000‐Hz sampling frequency. The peak torque during the MVIC and MVCC, and the knee angle associated with the peak angle during the MVCC were identified with a customized RStudio (Version 4.4). The best contraction for each time point was considered for the analysis.

### 2.5 Statistical Analysis

JASP (Version 0,14, JASP Team 2020; University of Amsterdam) was used for all statistical analyses. The Shapiro‐Wilk and Mauchly's tests were used to check the data normality and sphericity, respectively. A Greenhouse‐Geisser correction was applied if a non‐sphericity was observed. The raw values of MVIC, MVCC, MVCC optimal angle, and SWV were analyzed in a two‐way (time × age) analysis of variance (ANOVA). For all parameters, time corresponded to the four tests: Pre, Post, Post30 min, and Post48 h, and age corresponded to boys versus men. Post‐hoc tests with Holm correction were conducted if significant main effects or interactions were constated. If a significant time effect was observed, similar ANOVA as for raw values were conducted on relative values to test the age effect. The partial eta‐squared (η_p_^2^) was reported as a measure of the effect size, considering: 0.01 = small effect, 0.06 = moderate effect, ≥ 0.14 = large effect. Cohen's d was also reported and considered as follows: < 0.5 = small, 0.5–1.2 = medium, > 1.2 = large. Student *t*‐tests were used to examine the difference in the weekly training volume between boys and men. To determine the reliability of the elastography and torque measurements, the intraclass correlation coefficient (ICC_(3.1)_) of Shrout & Fleiss was used to compare the mean values obtained by the two videos and the two trials of contraction (Shrout and Fleiss 1979). The level of statistical significance was fixed at *p* ≤ 0.05.

## 3 Results

The reliability of the SWV and torque measurements showed very good reliability with values > 0.9 (Table 2).

**TABLE 2:** Descriptive statistics.

Variable	Group	Pre	Post	Post 30 min	Post 48 h
SWV VL 70° (m.s^−1^)	Boys	2.35 ± 0.14	2.26 ± 0.17	2.33 ± 0.25	2.36 ± 0.13
ICC_3.1_ = 0.932 (0.837; 0.973)	Men	2.34 ± 0.24	2.30 ± 0.27	2.21 ± 0.21	2.33 ± 0.19
SWV VL 110° (m.s^−1^)	Boys	3.29 ± 0.28	3.10 ± 0.24	3.16 ± 0.23	3.26 ± 0.26
ICC_3.1_ = 0.941 (0.858; 0.976)	Men	3.29 ± 0.29	3.18 ± 0.37	3.06 ± 0.30	3.19 ± 0.37
SWV RF 70° (m.s^−1^)	Boys	2.35 ± 0.22	2.23 ± 0.30	2.32 ± 0.28	2.37 ± 0.21
ICC_3.1_ = 0.965 (0.914; 0.986)	Men	2.25 ± 0.18	2.12 ± 0.16	2.09 ± 0.23	2.30 ± 0.17
SWV RF 110 (m.s^−1^)	Boys	3.03 ± 0.42	2.74 ± 0.38	2.99 ± 0.37	3.02 ± 0.37
ICC_3.1_ = 0.959 (0.899; 0.983)	Men	3.20 ± 0.39	3.28 ± 0.57	3.13 ± 0.43	3.33 ± 0.49
SWV PT 70° (m.s^−1^)	Boys	6.51 ± 0.56	6.93 ± 0.75	6.97 ± 0.53	6.85 ± 0.79
ICC_3.1_ = 0.988 (0.970; 0.995)	Men	9.46 ± 1.56	9.73 ± 1.51	9.75 ± 1.56	9.82 ± 1.65
MVIC (N.m)	Boys	74.51 ± 21.66	72.82 ± 23.56	72.96 ± 21.55	79.32 ± 25.89
ICC_3.1_ = 0.994 (0.984; 0.998)	Men	250.32 ± 74.63	225.66 ± 63.95	227.15 ± 63.57	251.32 ± 71.77
MVCC (N.m)	Boys	70.40 ± 14.73	77.79 ± 21.94	70.38 ± 22.75	73.32 ± 21.42
ICC_3.1_ = 0.975 (0.937; 0.990)	Men	215 ± 66.42	211.94 ± 59.71	214.75 ± 63.28	235.68 ± 65.48
MVCC optimal angle (°)	Boys	85.13 ± 11.81	89.78 ± 6.70	90.02 ± 6.43	88.53 ± 10.96
ICC_3.1_ = 0.518 (0.109; 0.777)	Men	85.53 ± 7.47	88.43 ± 5.67	84.85 ± 5.88	85.53 ± 5.93

*Note:* Values are means ± standard deviation.

Abbreviations: ICC: intraclass correlation coefficient for Pre values; MVIC: Maximal voluntary isometric contraction torque; MVCC: Maximal voluntary concentric contraction torque; PT: Patellar tendon; RF: Rectus femoris; SWV: Shear‐wave velocity; VL: Vastus lateralis.

### 3.1 Shear Wave Velocity

**FIGURE 2:**
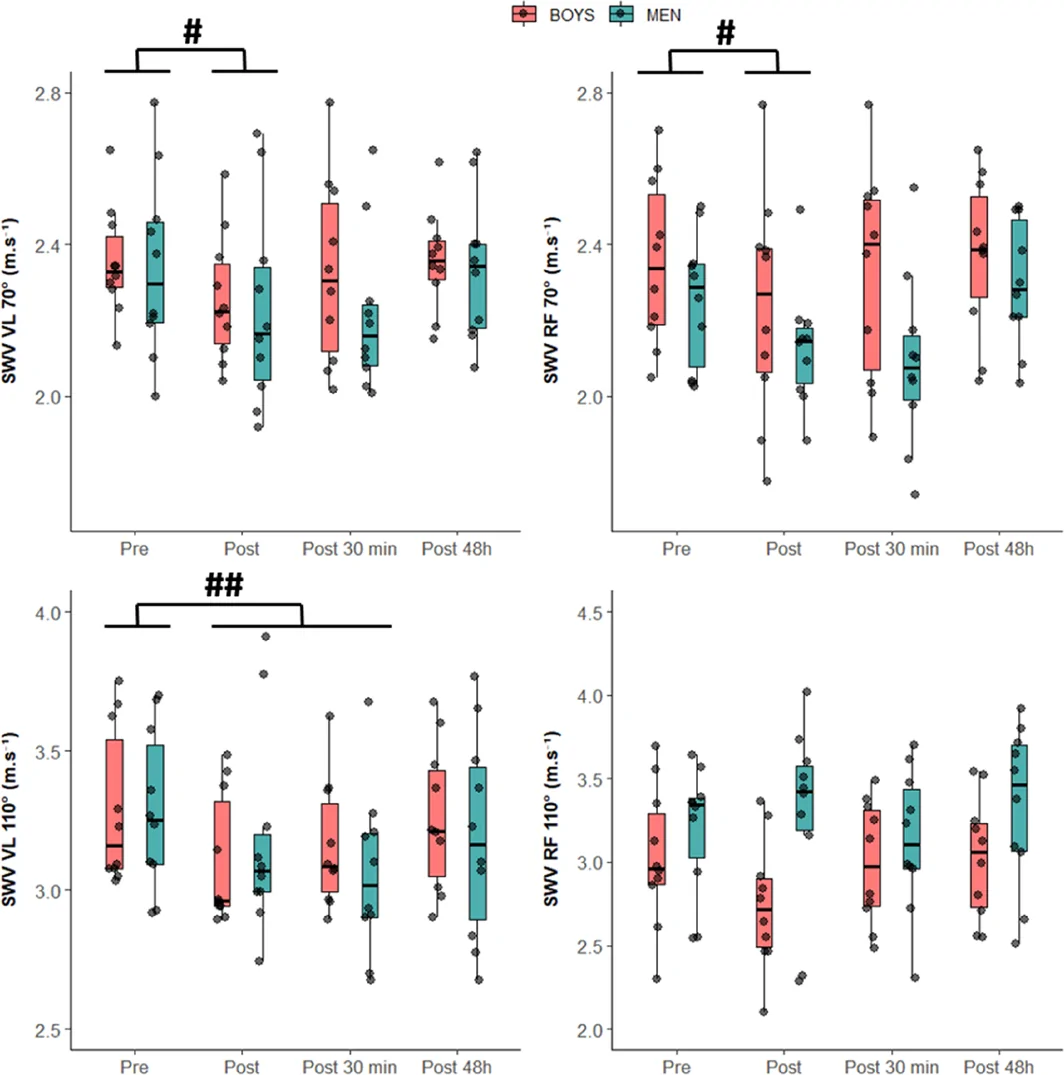
Shear wave velocity (SWV) time‐course for vastus lateralis (VL) and rectus femoris (RF). Significant differences with Pre are indicated with: # (*p* < 0.05) and ## (*p* < 0.01). A significant main time effect was observed for SWV VL 110° with a decrease at post and post30 min as compared to pre. Additionally, a significant time × age interaction was observed for SWV RF 110°.

A significant time effect was observed for SWV VL 70° (*p* = 0.007; ηp2 = 0.217) (Figure 2), revealing a decrease at Post as compared to Pre (*p* = 0.020; d = 0.499; Mean difference with 95% confidence interval (MD 95%CI): 0.102 (0.011; 0.194)). No age effect (*p* = 0.435) or interaction (*p* = 0.410) was observed for the SWV VL relative values at 70°.

A significant time effect was also observed for SWV VL 110° (*p* < 0.001; ηp2 = 0.313), revealing decrease at Post (*p* = 0.003; d = 0.489; MD 95%CI: 0.145 (0.035; 0.255)) and Post30 min (*p* < 0.001; d = 0.605; MD 95%CI: 0.180 (0.070; 0.290) as compared to Pre. No age effect (*p* = 0.556) or interaction (*p* = 0.145) was observed for the SWV VL relative values at 110°.

A significant time effect was observed for SWV RF 70° (*p* < 0.001; ηp2 = 0.260), revealing a decrease as compared to Pre at Post (*p* = 0.022; d = 0.528; MD 95%CI: 0.118 (0.006; 0.230)). No age effect (*p* = 0.626) or interaction (*p* = 0.132) was observed for the SWV RF relative values at 70°

A significant time × age interaction was observed for SWV RF 110° (*p* = 0.034; ηp2 = 0.148). The post‐hoc tests did not reveal any significant pairwise comparisons. For the SWV RF relative values at 110°, a significant time × age interaction was observed (*p* = 0.036; ηp2 = 0.169), but the post‐hoc tests did not reveal any significant difference between boys and men.

**FIGURE 3:**
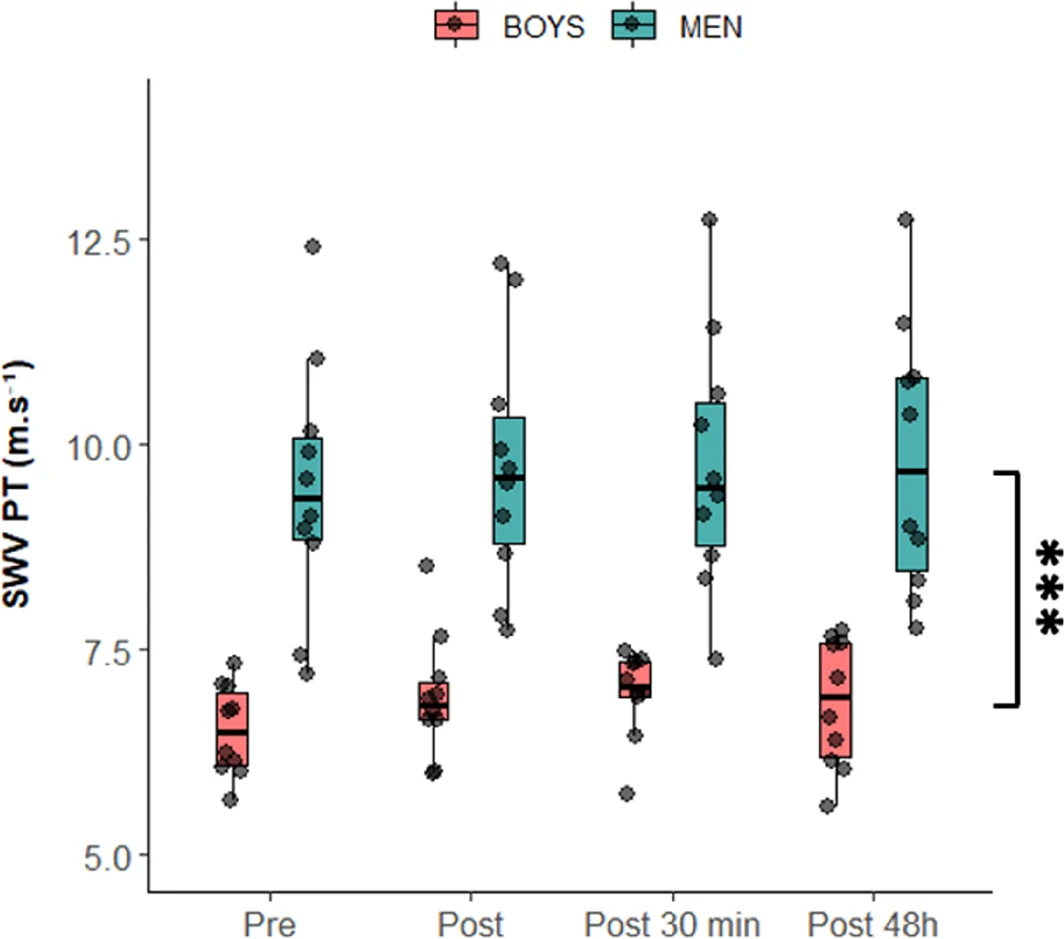
Patellar tendon (PT) shear wave velocity (SWV) time‐course. A significant main age effect was observed (*p* < 0.001).

No significant time effect was observed for SWV PT (*p* = 0.090) (Figure 3). However, a significant age effect was observed (*p* < 0.001; ηp2 = 0.648), revealing lower values for boys than for men, whatever the time point (*d* = −2.378; MD 95%CI: −2.872 (−3.921; −1.823)).

### 3.2 Torque

**FIGURE 4:**
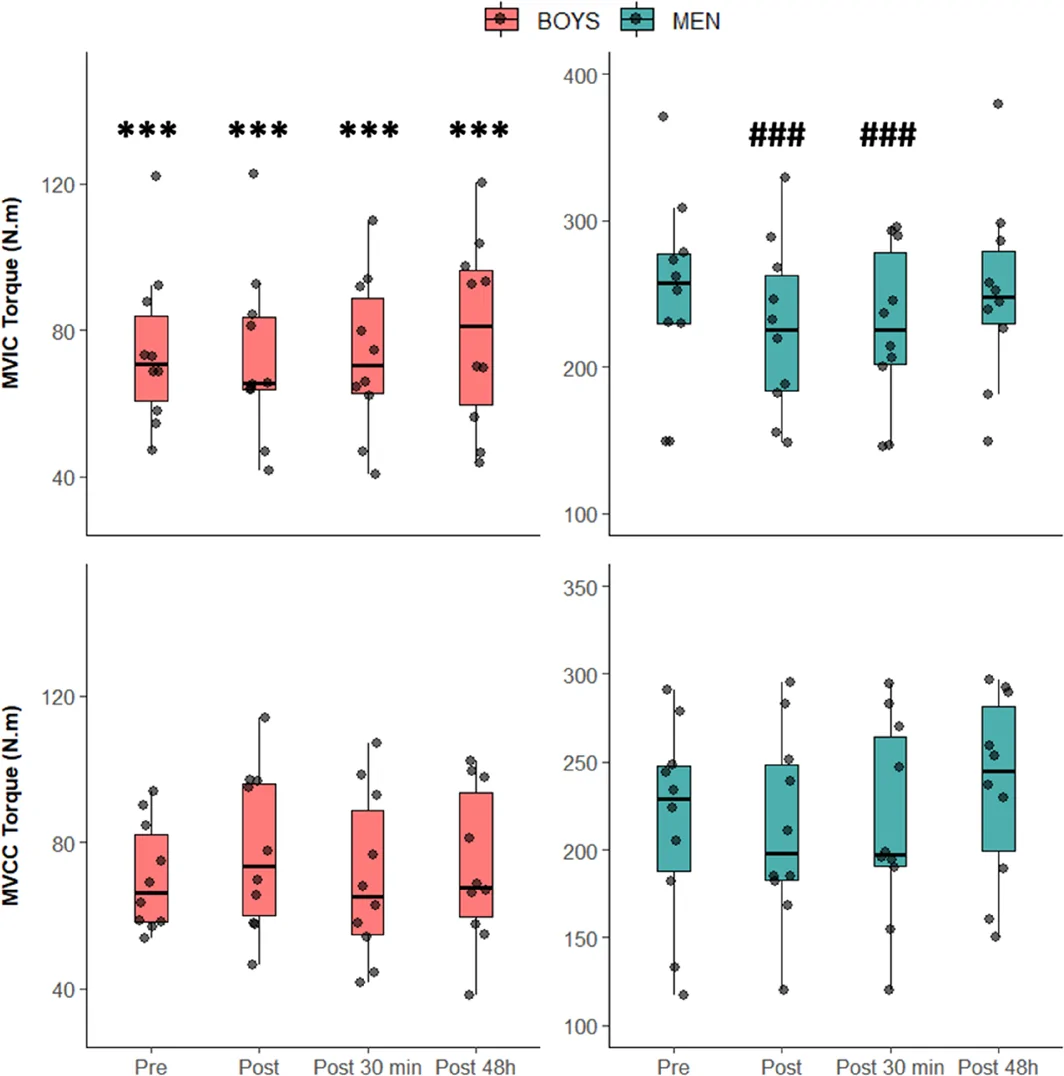
Maximal voluntary isometric (MVIC) and concentric (MVCC) torque time‐course. Significant differences with pre are indicated with: ### (*p* < 0.001). Significant differences between boys and men are indicated with: *** (*p* < 0.001). A significant main age effect was observed for MVCC.

A significant time × age interaction was observed for MVIC Torque (*p* = 0.004; ηp2 = 0.215) (Figure 4). Across all time points, boys exhibited lower MVIC torque values than men (*p* < 0.001). No significant change was observed in boys, while decreases were observed in men at Post (*p* < 0.001; d = 0.531; MD 95%CI: 24.664 (7.062; 42.266)) and Post30 min (*p* < 0.001, d = 0.499; (MD 95%CI): 23.165 (5.563; 40.767)) as compared to Pre.

No time effect (*p* = 0.108) or interaction (*p* = 0.85) was observed for MVCC torque. However, a significant age effect was observed (*p* < 0.001; ηp2 = 0.766), revealing lower values for boys than for men (*d* = −3.487; MD 95%CI: −146.581 (−183.541; −109.621).

### 3.3 MVCC Optimal Angle

No significant effect was observed for the MVCC optimal angle (Time *p* = 0.387; Age *p* = 0.276, Interaction *p* = 0.554). The mean optimal angle was 89.7 ± 11.8 and 88.4 ± 5.7, for boys and men, respectively.

## 4 Discussion

The main aim of this study was to compare the changes in myotendinous mechanical properties induced by a fatiguing concentric exercise between men and prepubertal boys. It was hypothesized that tendon properties would remain unaffected by exercise. The findings confirmed our expectations. There were no differences between boys and men in myotendinous mechanical properties. Additionally, the concentric exercise did not impair tendon properties.

The SWV of VL and RF was assessed to identify the effects of exercise on the muscle's mechanical properties. The values decreased immediately after exercise for both muscles, regardless of age group. This may suggest an acute reduction in muscle stiffness following concentric exercise. In contrast, a previous study in adults reported no changes in muscle SWV properties following repeated concentric contractions of the elbow flexors (Lacourpaille et al. 2017). However, in that study, the exercise consisted of three sets of 10 contractions at 120°.s^−1^ likely generating lower torque and involving a shorter exercise duration than in the present study. Additionally, the previous study implied the elbow flexor muscles, while the present study focused on the knee extensors. Conversely, the present results were consistent with previous observations in VL after knee extensors isometric contractions (Siracusa et al. 2019; Chalchat et al. 2020). In previous studies, the decrease in SWV was attributed to a reduced viscoelasticity of connective tissues, likely due to increased muscle temperature (Warren et al. 1971; Strickler et al. 1990). Given that concentric contractions are known to enhance muscle blood perfusions, this reinforces the hypothesis of temperature‐induced reduction in SWV (Laaksonen et al. 2003). Although the exact mechanisms behind this decline remain unclear, these changes could have functional implications, such as impaired force transmission (Chalchat et al. 2020; Morel et al. 2019) or an increased electromechanical delay (Siracusa et al. 2019). However, this relationship has not yet been clearly established (Lacourpaille et al. 2016).

The exercise did not induce modifications of the SWV PT, suggesting that there was no change in its mechanical properties. It is reasonable to suppose that the shortening of the myotendinous complex, characterizing the concentric contraction mode, in addition to the low torque (as opposed to eccentric contractions), may lead to a reduced mechanical loading of the tendon tissue. In contrast, in previous studies in adults, it has been demonstrated that eccentric and isometric contractions that induce a greater demand on tendon tissues could affect acutely the tendon mechanical properties (Kubo et al. 2001; Obst et al. 2016; Yin et al. 2014). Altogether, these results suggested that concentric exercise effects on the myotendinous mechanical properties in boys and men would be trivial. Thus, this exercise modality could be recommended to minimize mechanical properties impairment on the myotendinous structures.

No decreases in MVIC and MVCC were observed in boys after the fatiguing exercise. This differs from the current literature, which generally reports a decrease in torque following fatiguing exercises. In the present study, torque measurements after exercise were performed following SWV data collection. Therefore, it is reasonable to assume that the time required to perform these measurements allowed boys to recover (Ratel et al. 2015). This recovery period could also explain the lack of MVCC decline in men. A previous study has shown that 3 minutes of passive recovery are sufficient to restore maximal concentric torque production after fatiguing exercise (Cometti et al. 2011). In contrast, the results in men for MVIC are consistent with the literature. MVIC decreased immediately after exercise and remained reduced at Post30 min but returned to baseline at Post48 h. This pattern aligns with previous findings indicating that concentric contractions do not cause delayed torque loss (Dundon et al. 2008). This observation is coherent with the idea that delayed torque loss is a consequence of muscle damage, which concentric exercise is supposed not to induce. This interpretation confirms SWV's conclusions.

The results of this study have not shown differences between age groups in the myotendinous mechanical properties changes. This observation supports the idea that the difference between boys and men in the effects of exercise on myotendinous mechanical properties depends on the type of exercise. In fact, current literature shows that after eccentric or plyometric exercise, prepubertal boys have experienced less muscle damage than adults (Chen et al. 2014; Marginson et al. 2005). Conversely, a similar reduction in the stiffness of the plantar flexor has been observed between men and prepubertal boys after isometric repeated contractions (Piponnier et al. 2020). Recently, a study has demonstrated that prepubertal boys exhibited a similar increase in patellar tendon SWV following eccentric contractions, while only men experienced an increase in RF SWV (Chanel et al. 2026). The present study also suggests comparable immediate changes in myotendinous mechanical properties following concentric exercise. Therefore, these findings highlight the importance of diversifying the types of exercise studied to better understand how age influences fatigue development and to provide better recommendations for structuring physical activity, along with children's growth. From the present results, it is possible to recommend fostering the introduction of neuromuscular solicitations with concentric exercise, such as jumping or weightlifting exercises, to minimize the perturbations of their tendon mechanical properties and facilitate a high frequency of physical activity.

The present study has certain limitations that open up various research perspectives. First, these findings need to be supplemented with other parameters, such as electromechanical delay, to better understand the functional implications of the muscles' SWV decline observed immediately after exercise. Moreover, the initial SWV measurements were performed following the warm‐up, which may have influenced myotendinous mechanical properties. Future studies should control for this potential effect by also performing measurements prior to the warm‐up. In addition, the participants were regularly involved in competitive sports and were therefore accustomed to substantial loading of the myotendinous complex. Furthermore, habitual physical activity was not objectively quantified in the present study, which may have provided additional insights into the relationship between training status and exercise‐induced fatigue in children. Another limitations is that only boys and men were included, whereas fatigue responses may be influenced by sex. Finally, the present results should be replicated in a larger and more diverse sample to strengthen the generalizability the findings.

## 5 Conclusion

The present study compared the effects of concentric exercise on myotendinous mechanical properties between men and prepubertal boys. The results did not indicate differences between the two age groups, suggesting similar acute and delayed effects. Men and boys only exhibited a decrease in muscle SWV immediately after the exercise, while tendon mechanical properties were not affected by the concentric exercise. These findings suggest that concentric exercise may induce only limited acute alterations in myotendinous mechanical properties in both prepubertal boys and men. Given the limited sample size, these findings should be confirmed in larger and more diverse populations, together with complementary measurements, to strengthen the evidence and improve the generalizability of the results.

## Author Contributions

B.C. conceived and designed the experiment, performed the data collection, performed the statistical analysis, interpreted the data and wrote the first draft. N.B. conceived and designed the experiment, interpreted the data and reviewed the manuscript. C.C. N.B. conceived and designed the experiment, interpreted the data and reviewed the manuscript. All authors have read and approved the final version of the manuscript and agree with the order of presentation of the authors.

## Funding

The authors have nothing to report.

## Conflicts of Interest

The authors declare no conflicts of interest.

## Data Availability

The data that support the findings of this study are available from the corresponding author upon reasonable request.
